# Malformation of the Cortical Development Associated with Severe Clusters of Epileptic Seizures

**DOI:** 10.3390/vetsci10010007

**Published:** 2022-12-23

**Authors:** Aurora Cocchetto, Antonella Gallucci, Federica Biggio, Carlo Cantile

**Affiliations:** 1 San Marco Veterinary Clinic and Laboratory, Neurology and Neurosurgery Division, 35030 Veggiano, Italy; 2 Veterinary Neurological Centre “La Fenice”, 09047 Selargius, Italy; 3 Department of Veterinary Sciences, University of Pisa (PI), 56126 Pisa, Italy

**Keywords:** canine cortical dysplasia, feline lissencephaly, drug-resistant epilepsy

## Abstract

**Simple Summary:**

The malformation of the cortical development is a heterogeneous group of lesions, due to the neural migration and organization disorders, frequently associated with drug-resistant epilepsy in humans. Reports of epileptic seizure activity, due to the malformation of the cortical development in animals, as well as the descriptions of such neural malformations, are very rare. The aim of this case series is to describe the clinical presentation and neuropathological findings of three young animals with a malformation of the cortical development presented with a severe cluster of epileptic seizures that failed to improve with combined antiepileptic therapy, and were humanely euthanized. The first case described is a 3 month old mixed breed dog with a form of focal cortical dysplasia, the second case is a 21 day old Border Collie with a diffuse cortical dysplasia and the third case is a 2.5 month-old domestic cat with lissencephaly. Since, in pediatric neurology, these disorders are commonly associated with epilepsy, we suggest that although there is no direct evidence, these conditions, in our cases, may be accountable for epileptogenesis.

**Abstract:**

Three cases of the malformation of the cortical development are described: a mixed breed dog and a Border Collie pup with a focal and diffuse cortical dysplasia, respectively, and a kitten with lissencephaly. All cases presented with intractable epilepsy and were euthanized, due to the cluster of epileptic seizures. The gross examination at necropsy revealed the morphologic alteration of the telencephalic region in two cases. Histopathologically, a disorganization of the cortical lamination with the presence of megalic neurons, was found in the focal cortical dysplasia case. An altered organization of the white and gray matter, with a loss of the normal neuronal distribution and altered neurons, characterized the diffuse cortical dysplasia case. In the lissencephalic cat, there was no recognizable organization of the brain with areas of neuroglial tissue forming nodules in the leptomeningeal space. We strongly support the hypothesis that, as in humans, as well as in the veterinary patients, malformations of the cortical development could be the cause of refractory epilepsy.

## 1. Introduction

In human neurology, the malformation of the cortical development (MCD) is a heterogeneous group of lesions, due to the neuronal migration and organization disorders, which have been recognized, increasingly, in patients with drug-resistant epilepsy. A lack of a uniform, well-defined clinico-pathological classification of these disorders, is widely acknowledged in the epilepsy literature and hence attempts to classify the MCD to address the specific features of these disorders, including embryological, histopathological, imaging, and genetic aspects, have been proposed. “Cortical dysplasia” has been generically adopted to describe a variety of MCDs, including cerebral heterotopia and polymicrogyria. The term “focal cortical dysplasia” (FCD) indicates a type of MCD originally described by Taylor and colleagues [1], as one feature of which there is the presence of large dysplastic neurons and/or “balloon cells”. Minimal, microscopical abnormalities of the cortical architecture in patients with generalized epilepsy, are usually encompassed under the term “microdysgenesis”. Other forms of abnormal cortical development, including type I and II lissencephaly, polymicrogyria, and hemimegalencephaly, can be regarded as complex defects in the cell migration, in some instances, with distinct genetic etiologies.

Reports of epileptic seizure activity, due to a MCD in animals, as well as descriptions of such a neural malformation, are very rare. Epileptic seizures, in addition to other neurological signs, have been documented in animals affected with severe congenital anomalies, such as hydrocephalus, lissencephaly, and porencephaly, and occasionally in dogs with cerebrocortical lesions [2]. Reports of focal cortical dysplasia cases showing difficult to treat seizures, included a Pekingese dog with an associated necrotizing meningoenchephalitis [3], a Shi-Tzu with a concurrent porencephaly [4], a Golden Retriever with bilateral temporal cortical lesions [5], and a mixed breed dog with an associated granulomatous meningoenchepalitis [6]. Lissencephaly has been described as associated with epileptic seizure activity in few cases, that included four Lasha Apsos [7,8], a Pekingese [9], a mixed breed dog [10], an Australian Kelpie [11], a Shi-Tzu [12], a Wire-haired Fox Terrier [13] an Irish Setter [13], a domestic cat [14], and two litters of Korat cats [13]. Here we describe the clinical and neuropathological features of three cases of young animals with a MCD, affected with severe clusters of epileptic seizures, despite the starting of antiepileptic drugs (AEDs).

## 2. Case Description

Case 1 was a 3 month old mixed breed male dog, referred because severe epileptic seizure activity starting one month before, when the dog was found homeless by the present owner. The epileptic seizures were characterized by the tonic-clonic generalized activity, including autonomic signs. The frequency of the seizure activity was progressively increasing and, at presentation, was represented by a daily cluster of six to 10 epileptic seizures. The blood parameters and the cerebrospinal fluid (CSF) were normal, whilst the magnetic resonance imaging (MRI) was declined by the owner for financial issues. Phenobarbital therapy (3 mg/kg every 12 h), instituted after the neurological examination, was ineffective, despite its blood level in the therapeutic range of 25 μg/mL [15]. The dog was euthanized upon the owner’s request a few days later.

Case 2 was a 21 day old male Border Collie, presented underweight and underdeveloped with progressive neurological signs, including an altered mentation, circling, ataxia, and the sudden onset of epileptic cluster seizures (five in 24 h), the day before the presentation. The other littermates were normal, and no history of toxin exposure was reported. The blood parameters were normal, and the owner declined further investigation, including a MRI scan. The dog was treated with benzodiazepine (1 mg/kg per rectum, twice daily) and phenobarbital 2.5 mg/kg intramuscularly, every 8 h, without any improvement. The dog was humanely euthanized upon the owner’s request, ten days later due to the presence of seizures every 2–3 h.

Case 3 was a 2.5 month old domestic shorthaired male cat, presented with a rapidly progressive epileptic seizure activity, which in a few days reached the frequency of one episode every 15 min. The other littermates were normal, and no history of toxin exposure was reported. The diagnostic tools were not performed because of the owner constraint. The administration of benzodiazepine (1 mg/kg intravenously) and phenobarbital (up to 5 mg/kg IM) were ineffective, and the cat was euthanized upon the owner’s request, a few days later.

**Figure 1:**
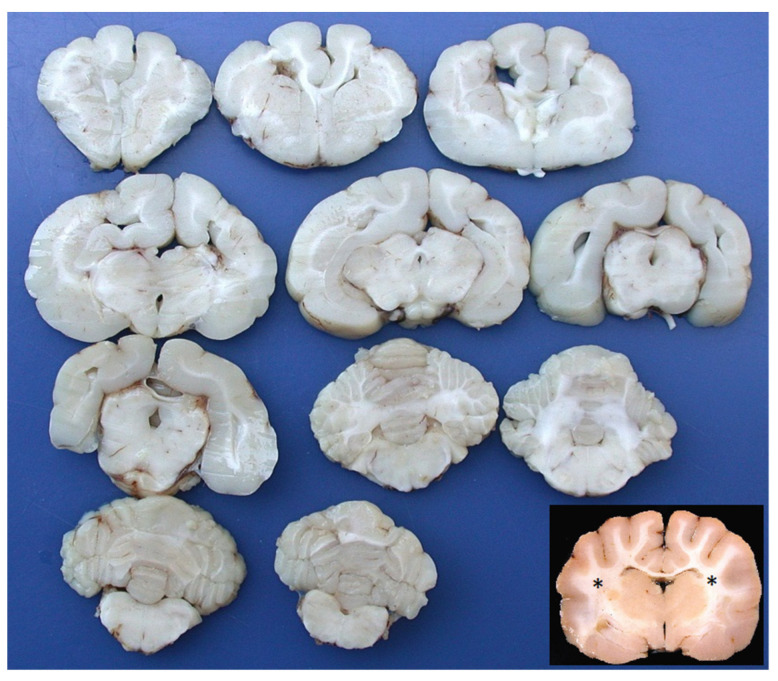
Cat brain. All transverse sections show the irregularly developed white matter of the telencephalon, with an absence of the corona radiata, an irregularly thickened cortex, a loss of sulci, and a broadening gyri, giving the brain a lissencephalic appearance. Basal nuclei, thalamus, brain stem, and cerebellum were macroscopically unremarkable. Inset: normal cat brain; transverse section at the level of the thalamus, approximately the same level of the first section of the second row at the top left (asterisks = corona radiata).

The gross necropsy findings were unremarkable in all the animals, except for the brains of cases 1 and 3, which showed anomalies of the telencephalic regions. The brains and samples of the major organs were fixed in a phosphate-buffered 4% formalin solution and then routinely processed for histology. In case 1, the transverse sections of the telencephalon showed a loss of the normal distinction between the gray and white matter, shallow sulci, and a mild enlargement of the ventricular cavities. The other encephalic areas were grossly normal. The cat brain (case 3) showed a bilateral and almost symmetrical anomaly of the cortical hemispheres. The gyri and sulci appeared roughly developed, giving a bumpy appearance of the cortical surface. The transverse sections of the fixed brain showed the absence of the corona radiata, an irregularly thickened cortex, a loss of sulci, and a broadening gyri. The gray-white matter junction was preserved but had an irregular appearance. The basal nuclei, thalamus, brain stem, and cerebellum were macroscopically normal (Figure 1).

Five micron-thick tissue sections were stained with hematoxylin and eosin (HE), Luxol fast blue (LFB), and Bielchowsky silver stain. 

The selected sections were stained immunohistochemically (IHC) with antibodies against the glial fibrillary acidic protein (GFAP, 1:1000; DakoCytomation, Carpinteria, CA, USA), and phosphorylated neurofilaments (clone 2F11, 1:1500; DakoCytomation). The heat-induced epitope retrieval was performed for both markers. The primary antibodies were incubated overnight at 4 C and the immunoreactivity was detected by the avidin-biotin-peroxidase complex method (Vectastain^®^ Elite ABC-Peroxidase kit; Vector Laboratories, Inc., Burlingame, CA, USA), using 3,3′-diaminobenzidine as chromogen. The negative controls were obtained by omitting the primary antibody.

**Figure 2:**
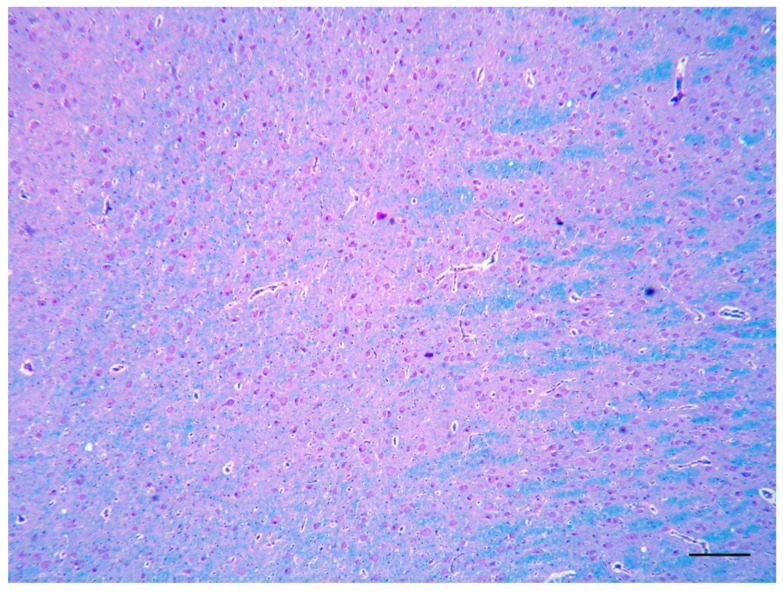
Telencephalon of dog 1. Numerous cytomegalic neurons are intermingled with normally conformed myelin tracts (LFB, bar = 250 µm).

**Figure 3:**
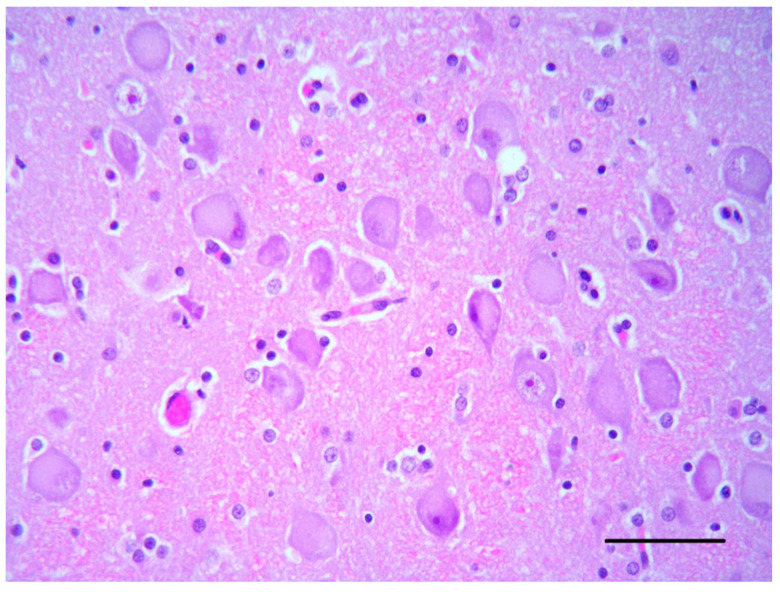
Cerebral cortex of dog 1. Many cortical neurons show an abnormal orientation, are enlarged with pale and eosinophilic cytoplasm and have eccentric nuclei (HE, bar = 50 µm).

**Figure 4:**
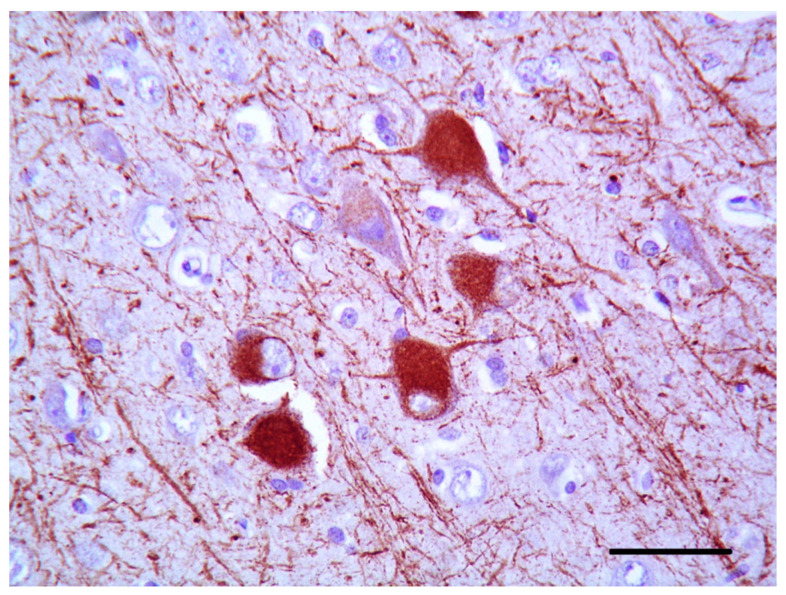
Cerebral cortex of dog 1. Megalic neurons’ cytoplasm strongly expresses phosphorylated neurofilaments (2F11 IHC, bar = 50 µm).

The histological examination of the brain of dog 1 revealed a marked disorganization of the cortical lamination with a presence of large randomly located cytomegalic neurons (Figure 2). The megalic neurons (“balloon cells”) had an abundant pale stained cytoplasm, often expressing the phosphorylated neurofilaments, and peripherally displaced nuclei (Figure 3 and Figure 4).

**Figure 5:**
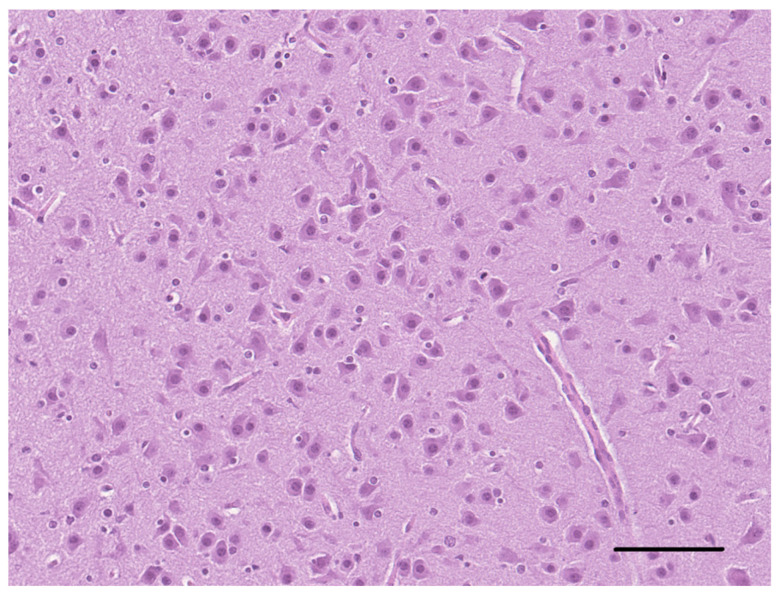
Cerebral cortex of dog 2. Neurons are haphazardly oriented and the distinction of the single cortical layers is not evident (HE, bar = 100 µm).

**Figure 6:**
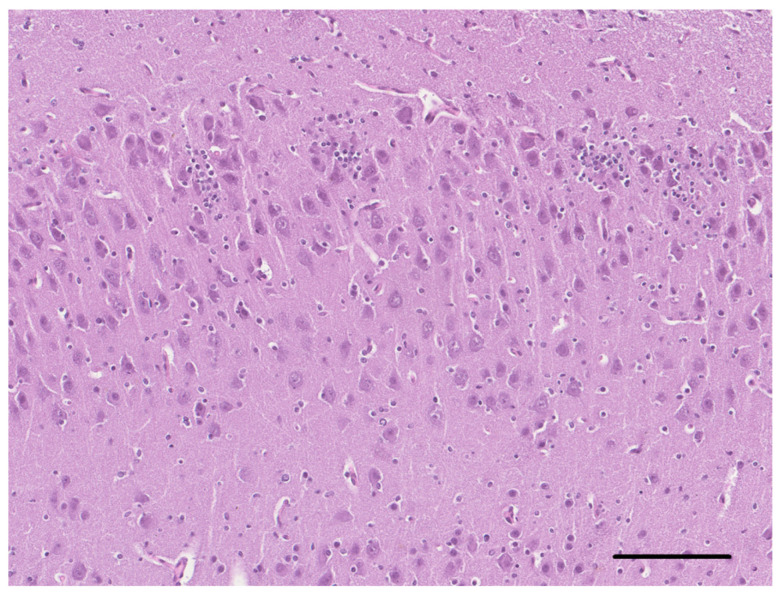
Hippocampus of dog 2. The pyramidal cell layer is disorganized and several nests of neuroblasts are intermingled (HE, bar = 125 µm).

**Figure 7:**
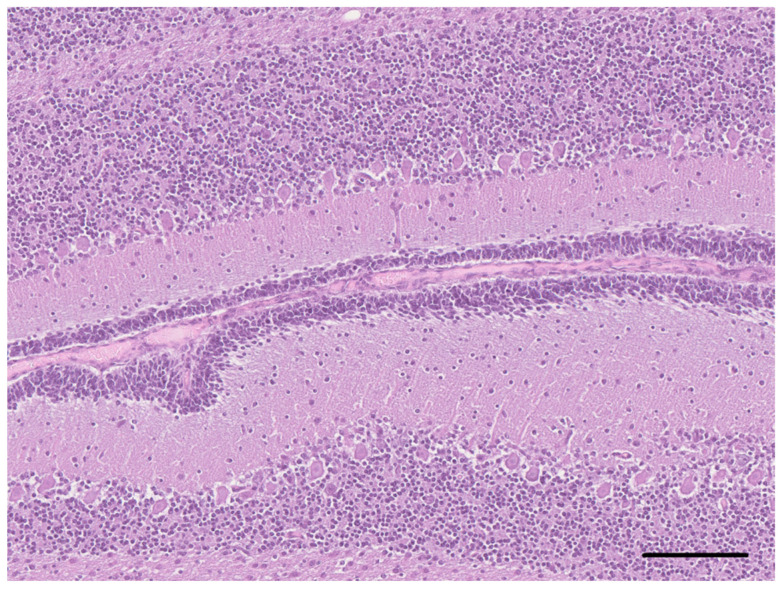
Cerebellum of dog 2. There is the presence of numerous immature neurons in the external granular layer of the cerebellar cortex (HE, bar = 125 µm).

On the histological examination of the brain of dog 2, an altered organization of the cerebrocortical layers characterized by the abnormally distributed, non-stratified neurons, also present in the subcortical white matter, was observed (Figure 5). The pyramidal neurons of the hippocampus also showed a disorganized distribution (Figure 6). Occasionally, the neurons in the midbrain showed multiple cytoplasmic vacuolations. In the periventricular areas, there were extensive aggregates of the neuroblasts and the presence of numerous immature neurons in the external granular layer of the cerebellum (Figure 7). Almost all ectopic neurons and numerous neurons located in the cerebral cortex were enlarged with abundant weakly eosinophilic cytoplasmic and irregular cytoplasmic profiles. The white matter morphology and the oligodendrocytes density were unremarkable in all brain areas.

**Figure 8:**
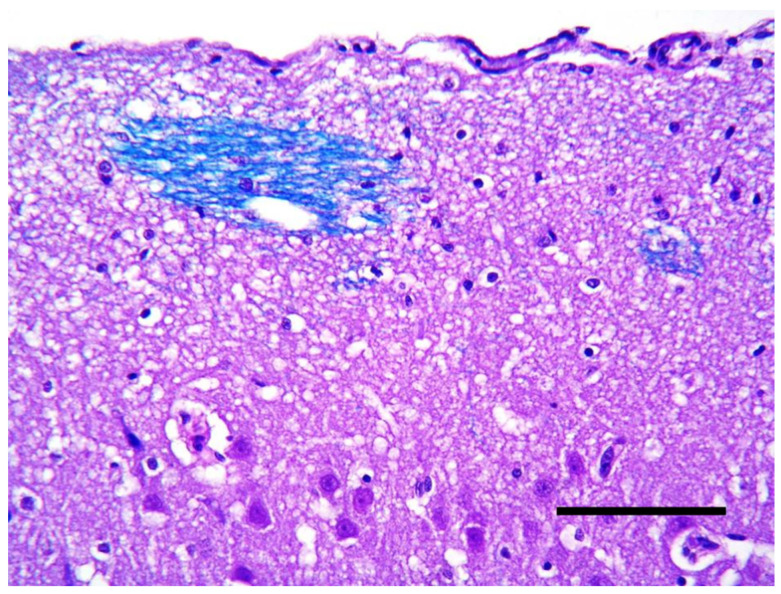
Cerebral cortex of the cat. Thin white matter tracts intermingled with the most superficial cerebral cortex (LFB, bar = 150 µm).

**Figure 9:**
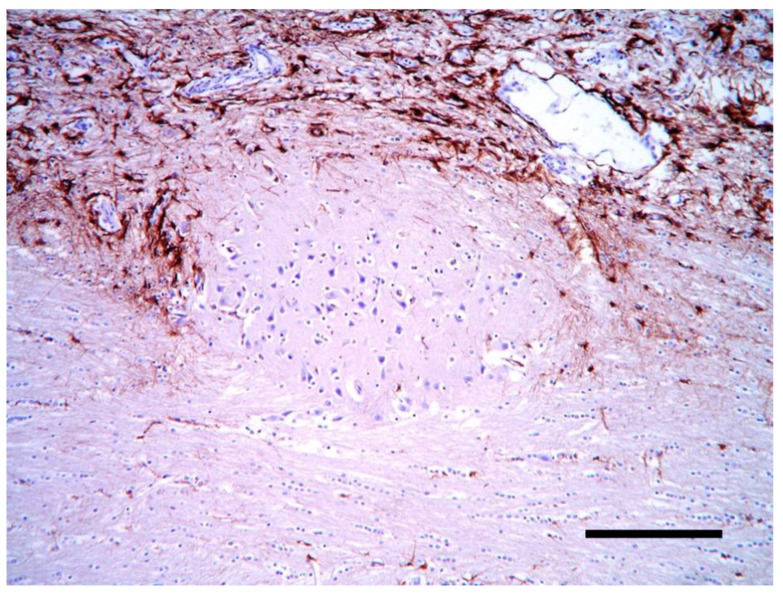
Cerebral cortex of the cat. A heterotopic cluster of neural cells is surrounded by astrocytes and prominent vascular structures (GFAP IHC, bar = 200 µm).

Histologically, the cortical architecture of the cat brain (case 3) was highly disordered with no recognizable lamination and no preservation of the molecular layer. Often, thin white matter tracts intermingled with the most superficial cortex (Figure 8). Multifocally, there were areas of neuroglial tissue extending over the cortical surface, forming nodules of disorganized cortex in the leptomeningeal space. Within the nodules, the immunohistochemistry for GFAP showed a disorganized collection of the reactive astrocytes (Figure 9). 

The other areas of the central nervous system (CNS) and the samples of major organs were unremarkable in all cases.

## 3. Discussion

In all cases, the morphological features of the observed lesions were indicative of different forms of a MCD. The histopathological pattern of case 1 was consistent with a form of cortical dysplasia of Taylor type IIb [16]. This entity is characterized by a massive spillover of abnormal neurons of the cortical-white matter junction, the laminar replacement of megalic and dysmorphic neurons (“balloon cells”) and well-preserved white matter [17]. Taylor-type IIb focal cortical dysplasia (FCD) has not been reported in the veterinary literature up until now.

FCD is a recognized condition in humans and, since its first description, it was considered a cause of medically intractable epilepsy [18]. The actual incidence is unknown, but among people with pharmacoresistant epileptic seizures, especially children, it accounts for most neocortical epilepsy cases [19]. FCD is classified in type I, II, and III depending on the morphology, the expression of the proteins and the association with other brain lesions [19]. Human MRI in FCD I usually shows no visible changes, while in FCD II, the focal cortical thickening, fuzziness between the gray and white matter, the white matter hyperintensity on T2 weighted images (T2WI) and fluid attenuated inversion recovery (FLAIR), the widened gyri, and abnormal sulci can be observed [20]. There are some available techniques used for the precise detection of the FCD lesions in human medicine, which show a good performance, mostly based on automated methods [20]. Reports of the MRI appearance of cortical dysplasia are lacking in veterinary medicine and only one study reported the MRI of a Golden Retriever with a bilaterally symmetrical cortical dysplasia that revealed symmetrical hippocampal and periventricular hyperintensities on T2 weighted images [5].

In humans the standard treatment is the surgical resection of the lesion [19] where, especially in type II, FCD has been associated with better seizure outcomes [18]. The absence of a visible lesion on MRI is one of the greatest challenges in epilepsy surgery and has led to an increase in invasive electroencephalography (EEG) studies [21].

In recent years, single causative gene mutations have been found for human FCD, probably due to the involvement of genes encoding regulatory proteins within the mechanistic target of the rapamycin (mTOR) pathway [19].

Four cases of FCD have been described in veterinary medicine, up until now [3,4,5,6]. In all cases, including the dog in our study, the most common presentation was aggressive and refractory epilepsy, which was the cause of the request for euthanasia. In all of the previous cases, except one, the dysplasia was found concurrently with other brain lesions that were considered more likely to be the epileptogenic focus. Since it is challenging to determine the relative contribution of each disease in the case of multiple brain disorders, the concept of multifactorial epileptic causes perhaps should be considered. 

The lesions observed in the brain of dog 2 were consistent with a developing immature brain with multifocal areas of dysplastic telencephalic cortex. The remnants of the external granular layer may not be an abnormal finding, since in puppies, it may be observed approximately until 10 weeks of age [22]. The organization and formation of the white matter was comparable to the findings described in 1 month old puppies [23]. Cerebral cortical dysplasia, characterized in this case by the laminar disruption and dysmorphic neurons, can be responsible for the seizure activity because of the early development of excitatory neurotransmitter systems and the delayed development of inhibition, as demonstrated in the immature brain [24].

In case 3, a type II lissencephaly (“cobblestone cortex”) was diagnosed. 

In humans, type II lissencephaly results from the overmigration of neural cells beyond the external glial limitans, whilst type I is linked to a primitive neuronal migration failure to reach the cortical plate, typically seen in dogs, such as Lhasa Apsos [7,8] and others [9,10,11,12].

Only a few reports of lissencephaly are reported in cats [13,14] and although many genetic mutations are recognized for human lissencephaly [25,26,27], genetic mutations have been suspected in dogs but not yet described [12]. Non-genetic causes, such as hypoxia and hypoperfusion, an intrauterine viral infection and metabolic disorders, are reported instead [28].

Epileptic seizures have been usually recorded in young affected animals and they seem to be responsive to antiepileptic drugs, in most cases [9,10,11,12,13,14]. The cat in our study seemed to have intractable epilepsy, as documented in humans. Indeed 90% of children with lissencephaly develop seizures and they are mostly associated with intractable epilepsy [29]. Other clinical signs reported, both in humans and in veterinary patients, are motor disability, neuro-ophtalmological abnormalities, and behavioral changes, including difficulty learning. In dogs and cats, aggressiveness and vocalizations are also observed [12].

MRI usually can reveal a smooth appearance of the cerebral surface and a thick cortical grey matter with classical lissencephaly, sometimes associated with other abnormalities [12]. In human cobblestone lissencephaly, MRI can also reveal a white matter signal abnormality, an irregular nodular cortex, which is usually associated with other malformations [30].

Since in pediatric neurology, these disorders are commonly associated with epilepsy, we suggest that although there is no direct evidence, the MCDs in our cases may be accountable for epileptogenesis. All cases described in this manuscript showed severe clusters of epileptic seizures that failed to improve with combined antiepileptic therapy. Even if the antiepileptic treatment instituted in our patients could be improved, the worsening of the seizure’s frequency, lead us to consider the possibility of drug resistant epilepsy. However, we cannot exclude a better treatment response using combined AED. In fact, although the presence of a high incidence of drug resistance in human patients with a MCD, a period of drug responsiveness could be achieved in a small proportion of patients [31]. Furthermore, in pediatric patients, the AED pharmacokinetic may be different, compared to adults and the adjustment of doses or the frequency of administration may be useful. So, in the case of a suspected MCD, an attempt with an aggressive antiepileptic treatment is recommended. 

We suggest that—although rare—a MCD may be responsible for the development of epileptic seizure activity in young animals and that, at least in some instances, the application of MRI techniques might facilitate the in vivo identification of some cortical malformations in animals with epilepsy. 

## Data Availability

All study data are presented in the article.
